# Toosendanin Restrains Idiopathic Pulmonary Fibrosis by Inhibiting ZEB1/CTBP1 Interaction

**DOI:** 10.2174/1566524023666230501205149

**Published:** 2023-11-22

**Authors:** Xingbin Li, Zina Bai, Zhensheng Li, Jun Wang, Xixin Yan

**Affiliations:** 1 Department of Respiratory and Critical Care Medicine, The Second Hospital of Hebei Medical University, Shijiazhuang, Hebei, 050005, China;; 2 Department of Respiratory and Critical Care Medicine, Hebei Chest Hospital, Shijiazhuang, Hebei, 050041,China

**Keywords:** Idiopathic pulmonary fibrosis, Toosendanin, CTBP1, fibroblast, Drug Targets, ECM

## Abstract

***Background*:** Extensive deposition of extracellular matrix (ECM) in idiopathic pulmonary fibrosis (IPF) is due to hyperactivation and proliferation of pulmonary fibroblasts. However, the exact mechanism is not clear. Objective: This study focused on the role of CTBP1 in lung fibroblast function, elaborated its regulation mechanism, and analyzed the relationship between CTBP1 and ZEB1. Meanwhile, the anti-pulmonary fibrosis effect and its molecular mechanism of Toosendanin were studied.

***Methods*:** Human IPF fibroblast cell lines (LL-97A and LL-29) and normal fibroblast cell lines (LL-24) were cultured *in vitro*. The cells were stimulated with FCS, PDGF-BB, IGF-1, and TGF-β1, respectively. BrdU detected cell proliferation. The mRNA expression of CTBP1 and ZEB1 was detected by QRT-PCR. Western blotting was used to detect the expression of COL1A1, COL3A1, LN, FN, and α-SMA proteins. An animal model of pulmonary fibrosis was established to analyze the effects of CTBP1 silencing on pulmonary fibrosis and lung function in mice.

***Results*:** CTBP1 was up-regulated in IPF lung fibroblasts. Silencing CTBP1 inhibits growth factor-driven proliferation and activation of lung fibroblasts. Overexpression of CTBP1 promotes growth factor-driven proliferation and activation of lung fibroblasts. Silencing CTBP1 reduced the degree of pulmonary fibrosis in mice with pulmonary fibrosis. Western blot, CO-IP, and BrdU assays confirmed that CTBP1 interacts with ZEB1 and promotes the activation of lung fibroblasts. Toosendanin can inhibit the ZEB1/CTBP1protein interaction and further inhibit the progression of pulmonary fibrosis.

***Conclusion*:** CTBP1 can promote the activation and proliferation of lung fibroblasts through ZEB1. CTBP1 promotes lung fibroblast activation through ZEB1, thereby increasing excessive deposition of ECM and aggravating IPF. Toosendanin may be a potential treatment for pulmonary fibrosis. The results of this study provide a new basis for clarifying the molecular mechanism of pulmonary fibrosis and developing new therapeutic targets.

## 1 INTRODUCTION

Pulmonary fibrosis (PF) is a serious interstitial lung disease [1]. The main pathological features were early diffuse alveolitis, later pathological transformation of a large number of fibroblasts, and progressive abnormal accumulation of extracellular matrix (ECM) to replace normal lung tissue structure [2]. However, the pathogenesis remains unclear [3, 4]. During the occurrence of IPF, pulmonary fibroblast (FB) plays an important role in promoting IPF [5]. Under pathological conditions, FB secreted a large amount of extracellular matrix through its abnormal proliferation and transformation, thus directly triggering IPF [6]. In addition, FB can also act on epithelial cells and some inflammatory cells indirectly participate in the PF process. Therefore, more mechanisms of the role of lung fibroblasts in IPF need to be explored [7].

C-terminal-binding protein (CTBP) is an evolutionarily conserved transcription corepressor that regulates the expression of many genes [8]. In recent years, a large number of studies have shown that CTBP is highly expressed in lung cancer, prostate cancer, breast cancer, and other tumor tissues or cells [9-12]. CTBP is closely related to the occurrence, development, and prognosis of tumor. In the studies conducted on a variety of tumor cells [13-15], it has been found that CTBP overexpression can down-regulate E-cadherin [16]. In cholangiocarcinoma, CTBP1 protein expression was significantly up-regulated and negatively correlated with E-cadherin protein expression. CTBP1 may be involved in regulating and inhibiting the expression of E-cadherin protein in cholangiocarcinoma, thereby promoting tumor EMt [17]. However, the relationship between CTBP1 and pulmonary fibrosis is rarely reported.

ZEB1 is a member of the dizinc finger protein family and contains two zinc finger clusters, n-terminal and C-terminal clusters [18]. ZEB1 is an important nuclear transcription factor by binding to relevant DNA sequences and producing transcription [19]. At present, the ZEB1 gene is mainly focused on its role in promoting epithelial interstitial conversion [20]. It was found that zeB-1, a member of the dizinc finger protein family, was involved in the signaling of the transforming growth factor β1 (TGF-β1) pathway and regulated the activated transcription of TGF-β1 by binding to Smad protein [19]. Therefore, it is speculated that ZEB1 may be associated with fibrosis.

Toosendanin is a tetracyclic triterpene compound derived from toosendan fruit [21]. Toosendanin has certain toxicity and was mainly used in agriculture in the early stage. In recent years, *in vitro* studies have found that Toosendanin has certain inhibitory effects on breast cancer cells, liver cancer cells, human leukemia cells, and so forth [22, 23]. Toosendanin can inhibit the proliferation and activation of various cells to some extent. The proliferation and activation of fibroblasts are the main characteristics of pulmonary fibrosis, especially the high level in the progression of idiopathic pulmonary fibrosis [24]. Therefore, the purpose of this study was to observe the effects of Toosendanin on cell proliferation and activation in lung fibroblasts. To reveal the mechanism of Toosendanin inhibiting pulmonary fibrosis and provide a new reference for Toosendanin anti-pulmonary fibrosis research.

The purpose of this study was to investigate whether CTBP1 plays a role in the proliferation and activation of pulmonary fibrosis fibroblasts through ZEB1. In order to provide a new basis for clarifying the detailed molecular mechanism of fibroblast proliferation and activation and developing new therapeutic targets for pulmonary fibrosis.

## 2 MATERIALS AND METHODS

### 2.1 Cell Culture

The cells were resuspended in DMEM medium containing 10% FBS and 100 U/mL penicillomycin. Cells in the cryopreservation tube were transferred to the Petri dishes. The culture dish containing cells was placed in an incubator at 37°C with 5% CO_2_. When the cell density reached 80% ~ 90%, the cells were digested with 0.25% trypsin solution. The cells were seeded in 6-well plates with 1×10^5^ cells/well. After the cells were cultured for 24 h, the cells were randomly grouped.

### 2.2 Cell Transfection

The logarithmic growing cells were inoculated into 6-well plates with 2×10^5^ cells/well. After continued culture for 24 h, the cells were randomly divided into the blank control group (normal culture without transfection treatment), si-NC group (transfection si-NC), and siRNA group. siRNA was transfected into cells according to the Lipofectamine2000 transfection reagent instructions. The cells were cultured for 48 hours before subsequent experiments.

### 2.3 qRT-PCR

Using the RNA extraction kit of Japan TaKaRa company to extract RNA according to the instructions. Absorbance OD was measured by UV spectrophotometer, and RNA purity was detected by OD260:OD280. Reverse transcription was performed according to the instructions of the RNA PCR Ver3.0 (TaKaRa, Japan) kit. Nucleotide sequences were provided by GenBank. PCR reaction conditions: 95°C for 5 min; 95°C for 30s, 60°C for 30 s, 72°C for 30 s, 30 cycles; 72°C for 5 min. ZEB1: forward primer 5’-GGCAGAGAATGAGGGA GAAG-3’, reverse primer 5’-CTTCAGACACTTGCTCA CTACTC-3’. GAPDH: forward primer 5’-TATGATGA TATCAAGAGGGTAGT-3’, reverse primer 5’-TGTATC CAAACTCATTGTCATAC-3’. GAPDH was used as an internal reference, and the 2^-ΔΔCt^ method was used to calculate the relative mRNA expression.

### 2.4 Western Blot

The cells were collected and protein lysate containing phenylmethyl sulfonyl fluoride was added to precipitate the lysate cells on ice. The supernatant was collected centrifugally and its concentration was determined. According to the measured concentration, 30 μg proteins were added to the sample well and SDS-PAGE gel electrophoresis was performed. After electrophoresis, membrane transfer was carried out. A total of 5% skim milk powder was incubated at room temperature for 2 h, and the specific binding sites were removed. CTBP1 (1: 1000), ZEB1 (1: 1000), and GAPDH (1: 2000) antibody diluent was added, respectively. It was incubated in a shaker overnight at 4°C. A specific secondary antibody diluent (1: 5000) was added and incubated for 1 h in a shaking bed at room temperature. ECL luminescent solution was applied evenly, and the protein bands were detected by the gel imaging system. The gray values of each protein band were analyzed by ImageJ software.

### 2.5 BrdU Experiment

At the end of cell treatment, BrdU solution was added. Incubate in an incubator for one hour. Cell fluid was discarded, cleaned with PBS, and fixed with formaldehyde. Sealed at room temperature for 2 h. BrdU primary antibody was incubated overnight at 4°C. Fluorescence secondary antibody was incubated at room temperature for 2 h. The proportion of BrdU-positive cells in each group (%) was calculated. At least 3 independent experiments were repeated for each BrdU experimental group.

### 2.6 Animal Model of Pulmonary Fibrosis

Male C57BL/6 mice aged 8 ~ 10 weeks, weighing 22 ~ 26g, were purchased from the Beijing Academy of Military Medical Sciences. According to the classical bleomycin-induced pulmonary fibrosis mouse model modeling method [25]. Specific implementation methods are as follows: On day 0, the anesthetized mice were weighed and recorded. The mice were fixed on the operating table and their necks were disinfected with 75% ethanol. A scalpel was used to make a vertical incision of about 1 cm in length on the neck of the mouse. Microscopic tweezers were used to separate tissue and expose the trachea. The syringe is inserted into the trachea through the tracheal cartilage annulus towards the heart. Then the normal saline solution of bleomycin corresponding to its body weight was slowly injected at a dose of 2 mg/kg. Immediately turn the animal upright and from side to side so that the solution is evenly distributed in the lungs. The blank control group was injected with the same amount of normal saline. After abdominal anesthesia, the mice were fixed on the rat plate in an upright position, and no. 9 was injected with a curved needle into the trachea and the adenovirus, or the viral vector without the packaged target gene (both 10^9^ pFU/0.1 ml). Animal experiments were approved by the Laboratory Animal Ethical and Welfare Committee of Hebei Medical University (No.: IACUC-Hebmu-2022015).

### 2.7 Immunofluorescence

The logarithmic growth phase cells were inoculated at a density of 1×10^4^ cells/well. When the cells adhered to the wall and grew to 70% fusion, they were starved in a serum-free medium for 24 h. PBS was cleaned 3 times, and paraformaldehyde was fixed for 30 min. TritonX-100 Broken film 20 min, 5% bovine serum albumin (BSA) was sealed at room temperature for 1 h. Subsequently, a primary antibody was added (1:200, Proteintech, USA). It was incubated at 4°C overnight, and washed with PBS for 3 times. Fluorescence secondary antibody (1: 1000, Invitrogen Company, USA) was added and incubated at room temperature for 1 h. After PBS cleaning, DAPI was stained and observed under a microscope.

### 2.8 Statistical Analysis

Data were analyzed by SPSS 17.0 statistical software. Measurement data were expressed as mean ± standard deviation. One-way ANOVA was used to compare the mean values between the subgroups and the control group. The independent sample T test was used for pairwise comparisons. *P* < 0.05 indicated a statistically significant difference.

## 3 RESULTS

### 3.1 CTBP1 Expression was Up-regulated in IPF Lung Fibroblasts

**Fig. (1):**
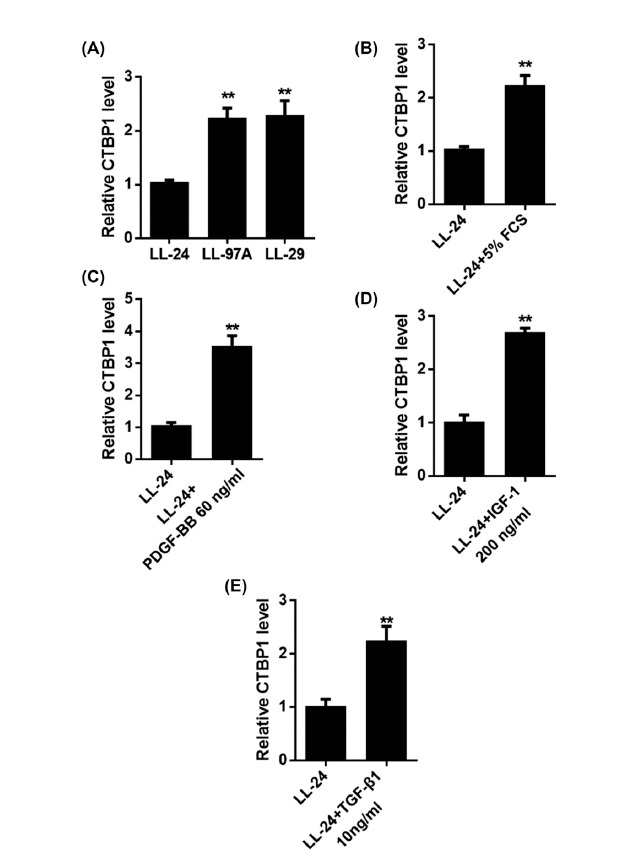
CTBP1 is upregulated in IPF lung fibroblasts. **A.** The expression of CTBP1 in human IPF fibroblast cell lines (LL-97A and LL-29) and normal human fibroblast cell line (LL-24) was detected by qRT-PCR. **B.** LL-24 cells were stimulated with fetal calf serum (5% FCS, treated 6h), and the expression of CTBP1 was detected by qRT-PCR. **C.** LL-24 cells were stimulated with platelet-derived growth factor-BB (60ng/ml PDGF-BB, treated 6 h), and the expression of CTBP1 was detected by qRT-PCR. **D.** LL-24 cells were stimulated with insulin-like growth factor 1 (IGF-1; 200 ng/ml, treated 6 h), and the expression of CTBP1 was detected by qRT-PCR. **E.** LL-24 cells were stimulated with transforming growth factor-β1 (TGF-β1; 10 ng/ml, treated 6 h), and the expression of CTBP1 was detected by qRT-PCR.

The expression of CTBP1 in human IPF fibroblasts (LL-97A and LL-29) and normal human fibroblasts (LL-24) was detected by QRT-PCR. Experimental results showed that CTBP1 was highly expressed in pulmonary fibrosis cell lines (Fig. **1A**). The expression of CTBP1 was detected by stimulating the cells with pulmonary fibrogenic cytokines (FCS, PDGF-BB, IGF-1, transforming growth factor –β1) to simulate the pulmonary fibrosis model. LL-24 cells were stimulated with cytokines and the expression of CTBP1 was detected by QRT-PCR. The experimental results showed high expression of CTBP1 after cytokine stimulation (Figs. **1B**-**E**). Through experiments, we found that CTBP1 expression was upregulated under the stimulation of cytokines. These results suggest that CTBP1 may play a role in promoting the progression of pulmonary fibrosis.

### 3.2 CTBP1 Attenuates TGF-β-induced Lung Fibroblast Activation

**Fig. (2):**
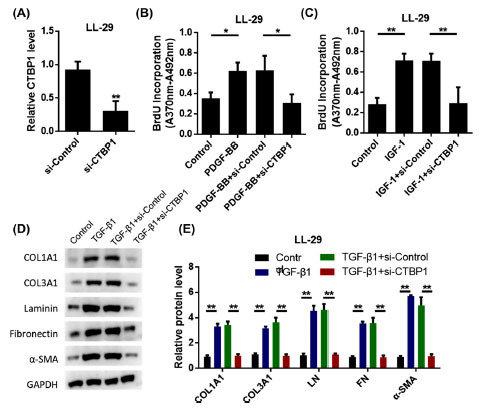
Silencing of CTBP1 inhibits growth factor-driven proliferation and activation of lung fibroblasts. **A.** LL-29 cells were transfected with si-CTBP1 or its negative control (si-control) to test the transfection efficiency. **B.** Cell proliferation was measured by BrdU incorporation method. **C.** Cell proliferation was measured by BrdU incorporation method. **D.** After stimulation with 10 ng/ml TGF-β1 for 6 h, western blot was used to detect 1a1 collagen (COL1A1), 3a1 collagen (COL3A1), laminin (LN), fibronectin (FN), α-smooth muscle actin (α-SMA) protein expression. **E.** Statistical results of protein expression.

Fibroblasts and myofibroblasts are major reflex cells that are activated after tissue injury and in patients with IPF, resulting in increased ECM production and enhanced migration potential. To investigate whether CTBP1 mediates functions related to fibroblast activation, fibroblast models treated with pulmonary fibrogenic cytokines *in vitro* were established. As shown in Fig. (**2A**), the expression of CTBP1 was significantly reduced after treatment with siRNA CTBP1. Results of the BrdU assay showed that the proliferation ability of LL-29 cells was significantly increased after PDGF-BB treatment. However, cell proliferation ability was significantly reduced in the intervention of CTBP1 silencing. It showed that silencing CTBP1 inhibited the proliferation of PDGF-BB-induced myoblast fibroblasts (Fig. **2B**). Similarly, siRNA CTBP1 can reduce the activation of IGF-1 on LL-29 cells (Fig. **2C**). Western blot results showed that TGF-β1 increased the main ECM components, including COL1A1, COL3A1, LN, FN, and α-SMA protein levels. When treated with siRNA CTBP1, their expression was significantly reduced (Figs. **2D**-**E**). These results suggest that CTBP1 can affect the proliferation and activation of fibroblasts.

**Fig. (3):**
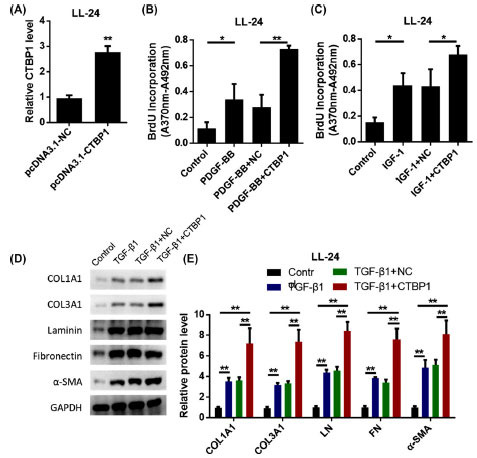
Overexpression of CTBP1 promotes growth factor-driven proliferation and activation of lung fibroblasts. **A.** LL-24 cells were transfected with pcDNA3.1-CTBP1 or its negative control (pcDNA3.1-NC) to test the transfection efficiency. **B.** Cell proliferation was measured by BrdU incorporation method. **C.** Cell proliferation was measured by BrdU incorporation method. **D.** After stimulation with 10 ng/ml TGF-β1 for 6 h, western blot was used to detect 1a1 collagen (COL1A1), 3a1 collagen (COL3A1), laminin (LN), fibronectin (FN), α-smooth muscle actin (α-SMA) protein expression. **E.** Statistical results of protein expression.

In addition, we also analyzed the effect of overexpression of CTBP1 on fibroblasts (Fig. **3A**). BrdU results showed that overexpression of CTBP1 enhanced fibroblast proliferation induced by PDGF-BB and IGF-1 (Figs. **3B**-**C**). In addition, in human IPF fibroblast cell line LL-24, overexpression of CTBP1 up-regulated protein levels of COL1A1, COL3A1, LN, FN, and α-SMA (Figs. **3D**-**E**). These results suggest that overexpression of CTBP1 promotes the proliferation and activation of lung fibroblasts driven by growth factors.

### 3.3 Silencing CTBP1 Reduced the Degree of Pulmonary Fibrosis in Mice with Pulmonary Fibrosis

**Fig. (4):**
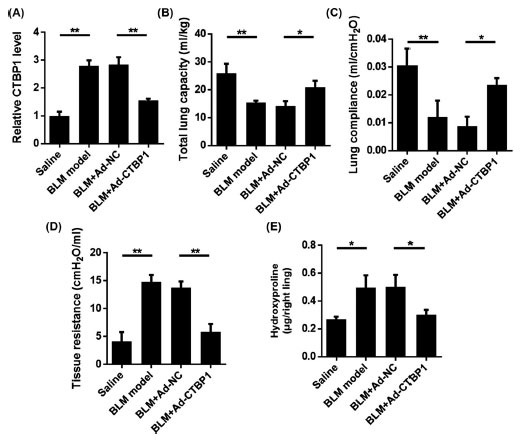
Silencing of CTBP1 attenuates the degree of pulmonary fibrosis in mice with pulmonary fibrosis. Mice were infected with Ad-CTBP1 or AD vector, and 2 days later, BLM (2 mg/kg) 50 μl saline was injected into the trachea to induce pulmonary fibrosis in mice. **A.** The expression level of CTBP1 was detected by qRT-PCR. **B.** 2 weeks after BLM stimulation, the total lung capacity of mice was measured. **C.** 2 weeks after BLM stimulation, mouse lung compliance was measured. **D.** 2 weeks after BLM stimulation, mouse tissue resistance was measured. **E.** Hydroxyproline assay kit for determination of hydroxyproline content in lung homogenate.

To further study the function of CTBP1, we constructed a mouse model of pulmonary fibrosis. Mice were infected with Ad-CTBP1 or Ad vector. Intratracheal injection of BLM (2 mg/kg) 50 μl normal saline induced pulmonary fibrosis in mice. The experiment was divided into the normal saline group and bleomycin group (BLM; N = 6), BLM + adenovirus vector (vector; N = 6), BLM + adenovirus CTBP1 (CTBP1; N = 6). Total lung capacity of mice was measured 2 weeks after BLM stimulation. The expression of CTBP1 in lung tissue showed that BLM treatment induced high expression of CTBP1. After silencing with adenovirus, the expression of CTBP1 decreased (Fig. **4A**). As shown in Fig. (**4B**), after BLM treatment, the total lung capacity of the mice was significantly reduced. However, the lung capacity of the mice increased with the silencing of CTBP1. Lung compliance decreased 2 weeks after BLM stimulation. However, lung compliance of mice was enhanced under the intervention of silent CTBP1 (Fig. **4C**). Tissue resistance was measured 2 weeks after BLM stimulation. Results showed that mice treated with adenovirus silencing CTBP1 showed improved lung tissue resistance (Fig. **4D**). Hydroxyproline in lung homogenate was determined by a hydroxyproline kit. Compared with bleomycin-treated mice, bleomycin-treated mice treated with adenovirus-silenced CTBP1 had reduced hydroxyproline content in lung tissues (Fig. **4E**).

### 3.4 Protein-protein Interaction Occurred Between CTBP1 and ZEB1

**Fig. (5):**
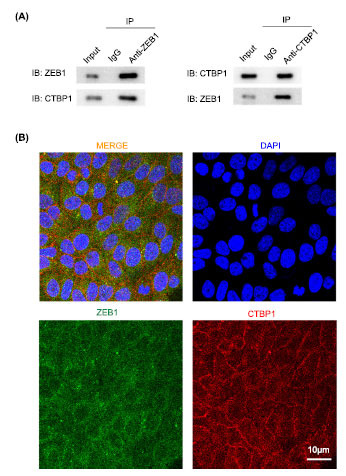
Protein-protein interaction between CTBP1 and ZEB1. **A.** CO-IP verifies that CTBP1 interacts with ZEB1. **B.** Double immunofluorescence staining confirmed intracellular co-localization of CTBP1 and ZEB1.

To further investigate the molecular mechanism by which CTBP1 affects fibroblast function, we predicted the interaction protein of CTBP1. The results showed that CTBP1 may interact with the ZEB1 protein. CO-IP verified the interaction between CTBP1 and ZEB1 (Fig. **5A**). Immunofluorescence double staining confirmed that protein interaction between CTBP1 and ZEB1 occurred in cells and was co-located in the cytoplasm (Fig. **5B**).

### 3.5 CTBP1 Promotes the Activation of Lung Fibroblasts Through ZEB1

**Fig. (6):**
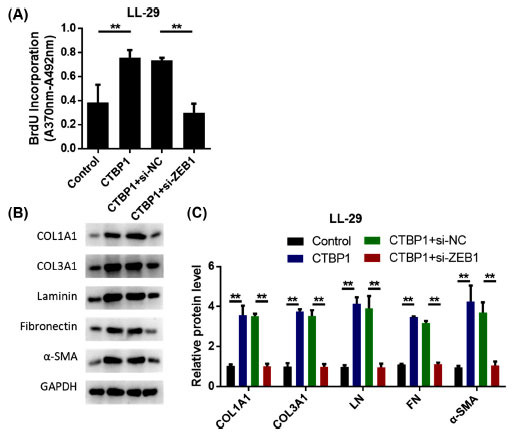
CTBP1 promotes lung fibroblast activation through ZEB1. **A.** Cell proliferation was measured by BrdU incorporation assay. **B.** 1a1 collagen (COL1A1), 3a1 collagen (COL3A1), laminin (LN), fibronectin (FN), α-smooth muscle actin (α-smooth muscle actin, α-SMA) were detected by western blot after stimulation with 10 ng/ml TGF-β1 for 6 h) expression. **C.** Statistical results of protein expression.

Cell proliferation was determined by the BrdU incorporation method. The results showed that overexpression of CTBP1 promoted the proliferation of fibroblasts, while simultaneous transfection of CTBP1 and siRNA ZEB reduced the proliferation of fibroblasts (Fig. **6A**). Western blot was used to detect the effects of CTBP1 and ZEB1 on the extracellular matrix. After 10 ng/ mL TGF-β1 stimulation for 6 h, the expression of 1A1 collagen (COL1A1), 3A1 collagen (COL3A1), laminin (LN), fibronectin (FN), and α-smooth muscle actin (α-SMA) proteins were detected by Western blot. After CTBP1 overexpression treatment, ECM-related protein expression was significantly increased. However, after siRNA ZEB1 intervention, ECM-related protein expression levels were significantly reduced (Figs. **6B**-**C**). The results showed that silencing ZEB1 inhibited the proliferation and activation of CTBP1-induced myoblast fibroblasts.

### 3.6 Toosendanin Inhibited the Proliferation and Activation of Lung Fibroblasts

**Fig. (7):**
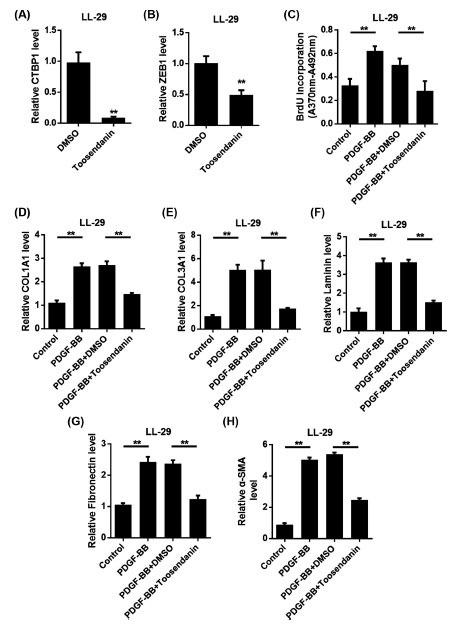
Toosendanin inhibits proliferation and activation of lung fibroblasts driven by growth factors. **A.** LL-29 cells were treated with Toosendanin to detect the expression of CTBP1. **B.** LL-29 cells were treated with Toosendanin to detect the expression of ZEB1. **C.** Cell proliferation was determined by BrdU incorporation. **D-F.** After PDGF-BB stimulation, qRT-PCR was used to detect the expression of collagen 1a1 (COL1A1), collagen 3a1 (COL3A1), laminin (LN), fibronectin (FN) and α-smooth muscle actin (α-SMA). Toosendanin treated cells for 24 hours.

Fibroblasts are activated after lung tissue injury and in patients with IPF, leading to increased ECM production and enhanced migration potential. In order to investigate whether Toosendanin can play a role in the treatment of pulmonary fibrosis through CTBP1/ZEB1, fibroblast models were established *in vitro*. As shown in Figs. (**7A** and **B**), the expressions of CTBP1 and ZEB1 were significantly reduced after Toosendanin treatment. BrdU assay showed that the proliferation ability of LL-29 cells was significantly increased after PDGF-BB treatment. But after Toosendanin’s intervention, the cell proliferation ability decreased significantly. These results indicated that Toosendanin inhibited the proliferation of myoblasts induced by PDGF-BB (Fig. **7C**). Further detection results of pulmonary fibrosis-related markers showed that PDGF-BB induction increased the expression levels of major ECM components, including COL1A1, COL3A1, LN, FN, and α-SMA. When treated with Toosendanin, their expression decreased significantly (Figs. **7D**-**H**). The above results showed that Toosendanin had the ability to affect the proliferation and activation of fibroblasts, and its effect was realized by inhibiting CTBP1/ZEB1.

## 4 DISCUSSION

As one of the most important cells in the process of pulmonary fibrosis, lung fibroblasts proliferate abnormally to form fibroblast foci and secrete ECM [26]. The deposition of ECM is a key event in the PF process and an important reason for the progress of PF [27]. In the process of pulmonary fibrosis, the combined influence of various factors, especially the stimulation of a large number of cytokines, will make FB continuously proliferate and transform into myofibroblast (MB) [28]. The most crucial factor is the transforming growth factor –β (TGF-β). TGF-β is currently considered to be one of the most fibrogenic cytokines [29]. By binding to the corresponding receptors, they stimulate the proliferation of fibroblasts and induce their transformation into MB [30]. Therefore, FB proliferation transformation is the main source of fibroblast foci. However, recent studies have shown [31] that cells divided into fibrotic foci in the middle of pulmonary fibrosis may be transformed from other lung cells, and some authors have proposed the concept of “epithelial-mesenchy-maltransition” (EMT) [32]. It is suggested that the transformation of alveolar epithelial cells into stromal cells is one of the important sources of local fibroblasts in the process of fibrosis.

In recent years, many cytokines have been widely used in the study of pulmonary fibrosis models *in vitro* due to their strong fibrogenic effects [33]. However, most of the research is limited to single cytokine stimulation. In this study, a variety of fibrogenic factors were used to construct cell models to better simulate the process of pulmonary fibrosis. The results of this study showed that fibrogenic factor stimulation can induce the expression of CTBP1 in fibroblasts, further stimulate cell proliferation and promote cell activation.

CTBP1 is an evolutionarily conserved transcription corepressor [34-36]. A large number of studies have shown that CTBP is overexpressed in a variety of tumors. CTBP1 promotes tumorigenesis mainly by regulating the transcription of related genes, including promoting EMT, tumor metastasis and Warburg, *etc* [37]. However, the biological function of CTBP is not completely clear at present. EMT is an important cellular mechanism in normal embryonic development characterized by the loss of polarity of epithelial cells and the acquisition of mesenchymal characteristics. Yao *et al*. [15] showed that CTBP1 binds ZEB1 through the PxDLS motif and forms a transcription inhibition complex that inhibits the transcription of E-cadherin, a key EMT regulator. It has also been confirmed that E-cadherin is a target gene of CTBP. CTBP inhibited E-cadherin. Overexpression of ZEB1, a negative regulator of E-cadherin, and E-cadherin inhibition have been reported in several human tumors. High levels of ZEB1 and CTBP expression were associated with low levels of E-cadherin [17]. These results suggest that CTBP1 plays an important role in the ZEB1 overexpression of tumor cells. EMT plays an important role in the development of fibrosis. Epithelial cells can activate a series of signal transduction pathways under specific physiological or pathological conditions. Inhibition of epithelial-associated proteins (E-cadherin) expression in epithelial cells led to the increased expression of mesenchymal markers (Fibronectin and α-SMA) resulting in an invasive mesenchymal phenotype and more fibroblast-like cells [38]. In this study, CTBP1 was found to be a novel profibrotic factor in idiopathic pulmonary fibrosis. CTBP1 can promote the activation of lung fibroblasts and the deposition of extracellular matrix (ECM) such as collagen and fibronectin by enhancing TGF-β signaling, ultimately exacerbating the development of pulmonary fibrosis. ZEB1, located on human chromosome 10P11.22, is a key transcription factor in CTBP1-mediated EMT and its related functions [39]. ZEB1 can promote cell proliferation, migration, and collagen formation through EMT, and eventually induce fibrosis [40]. Studies have shown that ZEB1 is involved in the progression of fibrosis, and fructose promotes EMT through up-regulation of ZEB1 expression, thus inducing liver fibrosis [41]. Targeting the CTBP1-ZEB1 complex may be a feasible strategy for the treatment of pulmonary fibrosis [42].

Fibroblasts are the most important source of ECM, so fibroblast-myofibroblast transformation (FMT) is the core event leading to pulmonary fibrosis [43]. A number of anti-pulmonary fibrosis drugs in the development stage that have been approved for the market have the function of inhibiting FMT. Nidanib and pirfenidone are approved for the clinical treatment of idiopathic pulmonary fibrosis, but they only slow the progression of the disease and do not relieve symptoms or reduce mortality. TCM has great potential in the field of new drug development [44, 45]. Many natural products from traditional Chinese medicine have been reported to have clear anti-pulmonary fibrosis functions. However, the unclear target of the natural product is one of the key factors limiting its patent medicine [46]. Clarifying the target of natural products is helpful to understand the molecular mechanism of their anti-pulmonary fibrosis. On this basis, the structure of the natural products was optimized according to the effective targets to improve the efficacy and avoid the toxic side effects, to make full use of Chinese herbal medicine resources to develop effective drugs to relieve pulmonary fibrosis. Fibroblast cell lines were used as the research object in this study. The results suggest that CTBP1 may be the target of Toosendanin. Toosendanin was found to inhibit the expression of various ECM markers. Therefore, this study revealed the target of Toosendanin in the treatment of pulmonary fibrosis. It is speculated that CTBP1/ZEB1 may be the protein target of Toosendanin playing the role of anti-pulmonary fibrosis.

## CONCLUSION

Our study preliminarily demonstrated the close association between ZEB1 and CTBP1 in pulmonary fibrosis on both animal and cellular levels. It is preliminarily confirmed that CTBP1 may be involved in the occurrence and development of pulmonary fibrosis. Perhaps by blocking its expression and reducing TGF-β1 activation, the progression of pulmonary fibrosis can be reduced or delayed. In addition, we found that Toosendanin plays an inhibitory role in pulmonary fibrosis by inhibiting CTBP1/ZEB1. It is believed that further research on it can provide a new target for the treatment of pulmonary fibrosis.

## Data Availability

The datasets used and/or analyzed in the current study are available from the corresponding author upon reasonable request.

## LIST OF ABBREVIATIONS

ECM: Extracellular Matrix
MB: Myofibroblast
IPF: Idiopathic Pulmonary Fibrosis
BSA: Bovine Serum Albumin
